# Supplementary figures and images for: Machine learning-based prediction of LDL cholesterol: performance evaluation and validation
Source: PeerJ. 2025 Apr 9;13:e19248. doi: 10.7717/peerj.19248 (PMC11992974; doi:10.7717/peerj.19248)

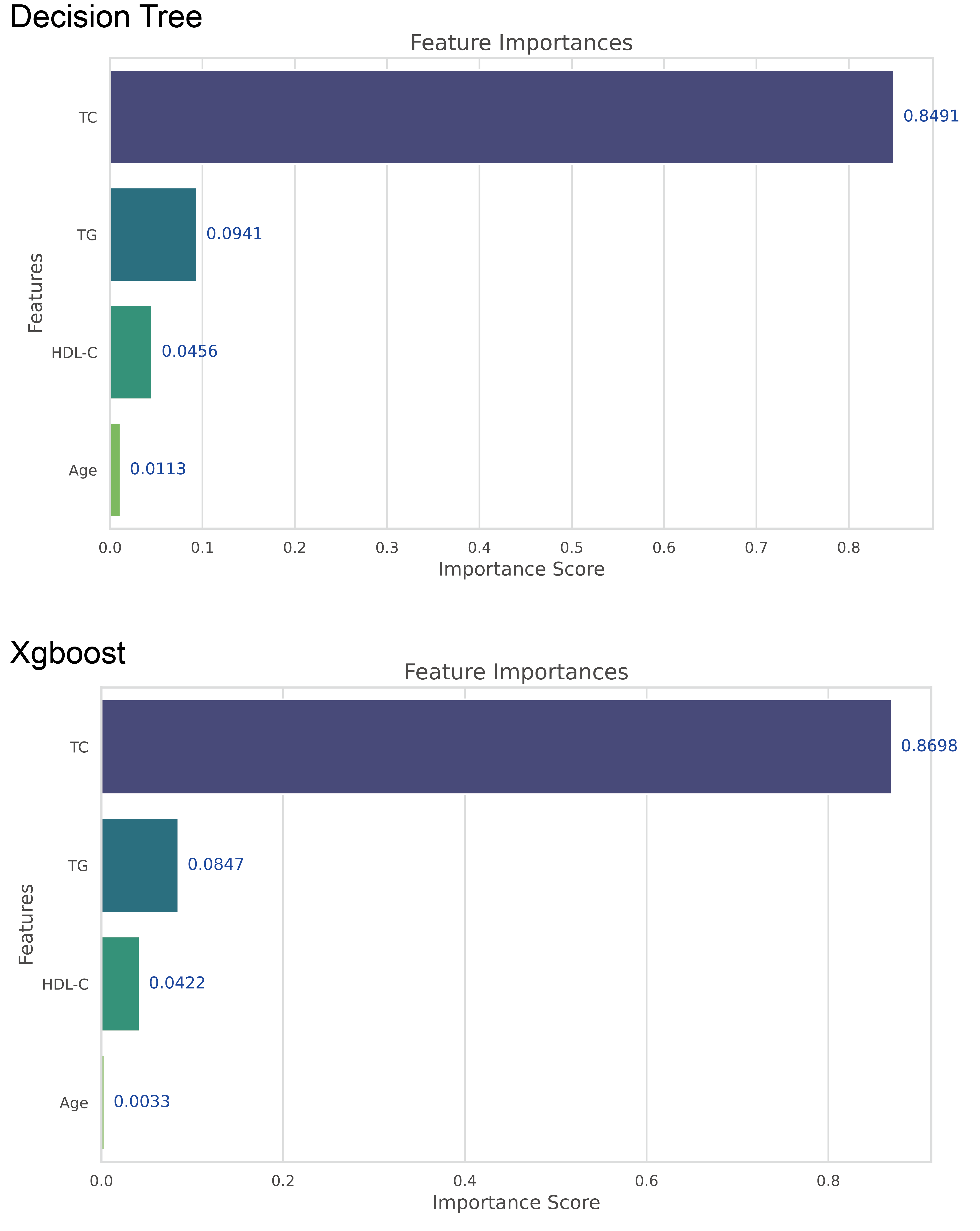

Supplement: Supplemental Information 2 [file peerj-13-19248-s002.png]
